# Microalbuminuria and Traditional Serum Biomarkers of Nephropathy among Diabetic Patients at Mbarara Regional Referral Hospital in South Western Uganda

**DOI:** 10.1155/2019/3534260

**Published:** 2019-12-16

**Authors:** Ritah Kiconco, Simon Peter Rugera, Gertrude N. Kiwanuka

**Affiliations:** ^1^Department of Medical Laboratory Science, Mbarara University of Science and Technology, Mbarara, Uganda; ^2^Department of Pathology and Diagnostics, Kampala International University Teaching Hospital, Bushenyi, Uganda; ^3^Department of Biochemistry, Mbarara University of Science and Technology, Mbarara, Uganda

## Abstract

**Background:**

Diabetic nephropathy (DN) is a common finding in diabetic patients. Microalbuminuria is the earliest clinical evidence of DN. Early detection of microalbuminuria is very important; it allows timely interventions to prevent progression to macroalbuminuria and later end-stage renal disease (ESRD).

**Objectives:**

To determine the prevalence of microalbuminuria in diabetic patients and establish its association with traditional serum renal markers in assessment of incipient nephropathy.

**Methods:**

This cross-sectional study involved 140 participants with diabetes mellitus (DM) attending the diabetic clinic of Mbarara Regional Referral Hospital. Questionnaires were used to obtain participant data after obtaining written informed consent. Data collected included: age, sex, level of education, history of smoking and alcohol consumption, hypertension, body mass index, family history, and duration of DM. Morning spot urine samples were collected from each participant and blood drawn for analysis of other renal markers. Urine microalbumin was determined quantitatively using immunoturbidity assay (Microalbumin kit, Mindray). Serum creatinine and uric acid and glucose levels were determined by spectrophotometric methods.

**Results:**

The overall prevalence of microalbuminuria was 22.9%. Using a simple and multiple linear regression model, serum creatinine (*β* = 0.010, 95% CI (0.005, 0.014), *P* = 0.0001) and glucose (*β* = 0.030, 95% CI (0.011, 0.048), *P* = 0.0017) levels were significantly associated with microalbuminuria. After adjusting for linearity, family history of DM was the only predictor of microalbuminuria (*β* = 0.275, 95% CI (0.043, 0.508), *P* = 0.002). Although microalbuminuria was weakly associated with eGFR (OR = 1.2, 95% CI (0.24, 5.96)), the relationship was not statistically significant (*P* = 0.824).

**Conclusion:**

The prevalence of microalbuminuria in patients with diabetes in this study was high. The study suggests the need to screen for microalbuminuria early to reduce the possible burden of ESRD. When serum creatinine is used as a renal function marker among diabetic patients, it should be combined with microalbuminuria for better assessment of incipient nephropathy.

## 1. Introduction

Diabetes mellitus (DM) is one of the most common endocrine disorder characterized by hyperglycemia [1]. According to the International Diabetes Federation, there were 366 million people with diabetes in 2011 and this figure is expected to increase to 500 million by 2030 [2]. Despite a great deal of research, the burden of disease is resting more heavily on tropical developing countries in Sub-Saharan Africa. Uganda, like the rest of the world, is experiencing an increasing prevalence of diabetes alongside other noncommunicable diseases [3].

Diabetic nephropathy (DN) on the other hand is a consequence of long-standing diabetes mellitus which is the leading cause of mortality among diabetic patients [4]. The disease is characterized by increased urinary albumin excretion in the absence of other renal diseases [5]. Microalbuminuria (MALB) is the appearance of albumin in urine ≥ 30 mg/day or 20 *μ*g/min and has been documented to be the earliest clinical evidence of diabetic nephropathy in DM patients [6]. Without specific intervention, patients with MALB have their urinary albumin excretions increased and eventually end up with end-stage renal disease [7]. Decreased glomerular filtration rate (GFR), elevated serum creatinine and uric acid levels, and electrolyte imbalances are key features in the laboratory diagnosis of DN here in Uganda hence termed traditional serum renal markers of nephropathy in this study.

At Mbarara Regional Referral Hospital's (MRRH) diabetic clinic, assessment of MALB is not a routine test. Usually, the clinic requests for the traditional serum renal biomarkers (serum uric acid, urea, and creatinine levels), electrolyte levels, and a blood slide test (to rule out malaria). Although these tests are requested for by the attending physicians, they are not based on evidence to benefit the diabetic patients directly. In addition, glycated hemoglobin (HbA1c) is requested to confirm the newly diagnosed patients. Given the Hospital's referral status, five to eight DM patients are admitted at the Hospital's Emergency Department on every clinic day if found to have high blood pressure. Additionally, another considerable proportion of patients with newly diagnosed DM do not have microalbumin measurements done at the diabetic clinic.

Whereas the above-mentioned traditional serum renal markers are implicated in the pathogenesis of renal disease, information from health personnel at MRRH diabetic clinic indicates that many patients cannot afford to pay for all these tests and so the tests are not done at the time of diabetes diagnosis. Lippi et al. [8] recently provided the definition of appropriateness in laboratory test requesting as “the Right test, using the Right method, at the Right time, to the Right patient, with the Right costs and for producing the Right outcome.” Thus, the objectives of this study were to determine the prevalence of microalbuminuria in diabetic patients, assess the factors associated with it, and establish its association with traditional serum renal markers (serum uric acid, urea, creatinine levels, sodium, potassium, chloride, and glucose) in assessment of incipient nephropathy.

## 2. Materials and Methods

### 2.1. Study Area

The study was carried out at the Diabetic Clinic of Mbarara Regional Referral Hospital (MRRH). The hospital is located in Mbarara district, South Western Uganda. The hospital is approximately 260 km from Kampala, Capital City.

### 2.2. Study Design and Population

This cross-sectional study involved a representative subset of the diabetic patients at MRRH. All patients enrolled in this study were already diagnosed with either Type 1 or Type 2 diabetes mellitus, receiving treatment from the diabetic clinic of MRRH, and had provided written informed consent. Pregnant women and patients with other medical kidney diseases were excluded from participating. One hundred forty diabetic patients who satisfied the inclusion criteria were enrolled into the study. Sampling of participants was done consecutively until the required sample size was obtained.

Pretested questionnaires were used to obtain participants' age, sex, year of diabetes diagnosis (recorded as duration with DM), and lifestyle risk factors, e.g., smoking and alcohol consumption. Blood pressure was measured on the day of enrolment into the study using a mercury sphygmomanometer with small (<21 cm) and normal (22-32 cm) cuff sizes on the left arm at the level of the heart while the patient was seated. Hypertension was defined as systolic blood pressure ≥ 140 and/or ≥90 mmHg or use of hypertensive medication. Participant's weight was measured using a weighing scale machine (Seca) making sure that the patient had no heavy clothing or shoes. Height was measured to the nearest 0.1 cm against a vertical wall. The participant's weight in kilograms and the square of their height in meters were used to calculate their body mass index (BMI). Diabetic nephropathy was determined using urine microalbumin, and serum levels of creatinine, uric acid, sodium, potassium, chloride, and glucose.

### 2.3. Urine and Blood Sample Collection and Storage

A random spot urine sample [9, 10] as indicated in the testing procedure using the Mindray MALB reagent kit was collected in a clean, dry screw cap urine container. Participants were given clear verbal and written instructions on how to collect the required midstream urine sample into the container. Using a vacutainer needle and holder, 4 mL of venepuncture blood was drawn into a red top vacutainer tube. Blood samples were centrifuged at 12,000 revolutions per minute to obtain serum. Serum was aliquoted into cryovial tubes and stored at -20°C until analysis at Kampala International University Teaching Hospital (KIUTH) Laboratory. Spot urine samples were aliquoted into cryotubes and frozen at -20°C. A single vial and cryotube for each participant was thawed once at 25°C and analysed. The random urine samples were analysed for microalbuminuria following the laboratory Standard Operating Procedures (SOPs).

System compatible reagent of the Microalbumin (MALB) kit from Mindray, for the quantitative determination of microalbumin, was used on a Mindray Chemistry Analyser (Shenzhen Mindray Bio-Medical Electronics Co., Ltd, BS 200, China). The same analyser was used to measure plasma creatinine, uric acid, and glucose levels. The procedures were calibrated and controlled using multisera calibrators and controls from Mindray of lot number 150115002 and 050215001, respectively. Serum electrolytes (sodium, potassium, and chloride) were analysed using a colorimeter (Environmental and Scientific Instruments Co., Digital Photo Colorimeter, India) at 630 nm, 500 nm, and 480 nm, respectively. All equipment was calibrated. Reference ranges used were those routinely used at KIUTH Laboratory. The types of albuminuria were defined as microalbuminuria (2-20 mg/dL), normoalbuminuria (<2 mg/dL), and macroalbuminuria (>20 mg/dL).

### 2.4. Data Analysis

Data were entered into Microsoft Excel 2013 and analysed using STATA version 12. Primary outcome variables were serum levels of creatinine, uric acid, sodium, potassium, chloride, and glucose; secondary outcome was diabetic nephropathy indicated by microalbuminuria. Independent variables included age, gender, and marital status, and tribe, level of education, family history of DM, BMI, and duration of DM, alcohol consumption, and smoking. Categorical variables were presented as percentages. Descriptive analysis, using statistical methods, was done. Estimated glomerular filtration rate was calculated as reported by [11]. Differences between proportions were assessed by Fisher's Exact Test. Logistic regression (odds ratio with 95% confidence intervals) was used to assess any association between microalbuminuria and estimated glomerular filtration rate (eGFR). The relationship between microalbuminuria and the traditional serum renal markers of diabetic nephropathy was assessed by linear regression (univariate and multivariate analysis). A *P* value of <0.05 was considered statistically significant.

## 3. Results

### 3.1. General Characteristics of the Study Participants

The general characteristics of the study participants are shown in Table 1. Of the 140 diabetic patients in this study, there were 95 (67.9%) females and 45 (32.1%) males. Most of the participants, 41 (29.3%), were aged between 45 and 54 years. Two districts, that is, Mbarara and Isingiro contributed majority of the participants, 105 (75%) and 20 (14.29%), respectively. Majority of the participants, 76 (54.3%), had received up to primary level education, 104 (74.3%) were married, 3 (2.1%) reported to be smoking, and 9 (6.4%) confirmed taking alcohol.

### 3.2. Clinical Characteristics of Diabetic Patients

Majority of the participants, 81 (57.9%), had normal blood pressure; their average systolic and diastolic pressure was 138.6mHg and 78.1 mmHg, respectively. One hundred and nine participants (77.9%) had a family history of DM, and the average duration of DM was 6.8 years. Seventy percent of study participants had normal BMI with the average being 24.4 kg/m^2^.

### 3.3. Estimated Laboratory Markers of Renal Function among Diabetic Patients

The mean values of the assessed biomarkers of renal function measured in serum and urine are shown in Table 2. The mean values of the traditional markers of nephropathy were within the laboratory reference intervals except for blood glucose which was elevated (9.3 mmol/L).

### 3.4. Estimated Categories of Albuminuria among Diabetic Patients

Majority of the 140 study participants, 32 (22.9%), had microalbuminuria, 107 (76.4%) normoalbuminuria, and 1 (0.7%) macroalbuminuria (Table 3). There was no statistically significant association between microalbuminuria and gender (*P* = 0.242) or with age (*P* = 0.941). Microalbuminuria was found in 19 (20%, 95% CI (13.03, 29.44)) females and 13 (28.9%, 95% CI (17.21, 44.26)) males. In addition, microalbuminuria was mostly detected among participants aged 45-54 years, 10 (24.4%, 95% CI (13.3, 40.4)), and the least affected age group was 18-34 years, 3 (23.1%, 95% CI (6.32, 57.2)).

### 3.5. Univariate and Multivariate Analysis for the Potential Association of Microalbuminuria

Linear regression analysis (Table 4) was performed with microalbuminuria levels as the dependent variable. A simple linear regression revealed serum creatinine (*β* = 0.010, 95% CI (0.005, 0.014), *P* = 0.0001), uric acid (*β* = 0.002, 95% CI (0.001, 0.003), *P* = 0.0071), and glucose (*β* = 0.030, 95% CI (0.011, 0.048), *P* = 0.0017) levels to have a positive correlation with microalbuminuria levels.

A unit increase in each biomarker corresponded to an increase in microalbuminuria levels. The rest of the variables, that is, sodium, potassium, and chloride levels did not have statistically significant correlation with microalbuminuria levels. A backward stepwise multiple linear regression analysis by elimination procedure was done to identify those markers that might be independently associated with microalbuminuria. Serum creatinine (*β* = 0.006, 95% CI (0.001, 0.011), *P* = 0.012) and glucose (*β* = 0.027, 95% CI (0.008, 0.0452), *P* = 0.005) levels were the only markers that seemed to correlate with microalbuminuria (Table 4).

Logistic regression showed a nonstatistically significant association between microalbuminuria and eGFR (OR = 1.2, 95% CI (0.24, 5.96) and *P* = 0.824).

## 4. Discussion

This cross-sectional study conducted at Mbarara, Regional Referral Hospital Diabetic Clinic in South Western Uganda provides the prevalence of microalbuminuria among diabetic patients, and its performance against traditional markers of renal function in assessment of incipient nephropathy. Microalbuminuria was detected in 22.9% of participants in this study. This prevalence is lower than what was reported in a study at Mulago National Referral Hospital in Uganda done by Muddu et al. [12]. The authors observed a prevalence of 54% microalbuminuria in 202 newly diagnosed diabetic patients, which was more than double than what we observed in the current study. Given that the participants in the current study were recruited over a period of one month compared to six months in the study at Mulago, the prevalence of microalbuminuria among diabetic patients at MRRH is relatively high. A longer study with a bigger sample size may possibly identify more diabetic patients with microalbuminuria at MRRH. Another difference is that the study at Mulago Hospital was done to assess microalbuminuria as a contributor to echocardiographic abnormalities among newly diagnosed diabetic patients and not a marker of nephropathy. Several epidemiological studies have reported the prevalence rates of MALB as ranging between 20% and 61% in patients with diabetes [7, 13, 14]. A study conducted in Senegal by Djiby et al. [15] to assess the prevalence of microalbuminuria and associated risk factors in a population of diabetics followed at the Marc Sankale Center of Dakar reported a prevalence rate of 27.14% among 221 participants. The prevalence of microalbuminuria of 27.14% is comparable to what was observed in the current study. Variations in the prevalence of microalbuminuria has been attributed to several factors like difference in populations, the definition of microalbuminuria by different laboratories, and the method of urine collection and of measurement of microalbuminuria [16]. Nevertheless, microalbuminuria in adults who formed the majority of our study participants is believed to be an early risk marker for nephropathy [17].

In this study, the definition of microalbuminuria of 2-20 mg/dL in a spot urine sample has been used elsewhere [6] and recommended by others [18, 19]. A study in India by Chowta et al. [20] reported a prevalence of microalbuminuria of 37% in 100 diabetic patients. In this same study, there were 20 (54.05%) males and 17 (45.95%) females with microalbuminuria. In comparison to our study, females, 19 (20%), dominated with microalbuminuria while males were only 13 (28.9%). Adjusting for age and sex did not show any statistically significant association with microalbuminuria in this study population. Lack of association between age and microalbuminuria may be because majority of our patients were above 30 years of age. The association has been reported to be significant in paediatric diabetic patients with Type 1 diabetes mellitus [21, 22] and age-at-onset specific. It has been suggested that sex hormones possibly play a role in incidence of microalbuminuria in young diabetic patients; however, this is significant for pubertal onset diabetes; prepubertal [23] and postpubertal onset of diabetes do not increase the risk of microalbuminuria [22]. We did not exclude female patients of reproductive age who may have been in their menstruation cycle from participation. Whereas some studies have indicated that contamination of urine sample with menstrual blood may give inaccurate results, in a large United States National Health and Nutritional Survey that involved 3,784 women aged 20-60 years with 290 (7.7%) having menses when blood was collected; urine protein measurement was not affected by menstruation [24]. While it is logical to expect false positive results during menstruation, menstrual blood would result in macroalbuminuria or proteinuria. We found macroalbuminuria in only one participant. Microalbuminuria was seen in only 24% of the participants, and they were older patients aged 45-54 years. Overall, our study population was comprised of mostly adult patients: 102 (72.8%) above 45 years of age. Of these, 61 (59.8%) were above 55 years of age. In view of this, it is unlikely that the possibility of some women being in menstrual cycle could have significantly affected the observed prevalence of microalbuminuria.

We observed a statistically significant correlation between microalbumin levels in urine and serum creatinine levels, serum uric acid levels, and serum glucose levels at univariate analysis. Microalbuminuria was not associated with sodium, potassium, and chloride levels. We used a backward stepwise multiple linear regression analysis by elimination procedure to identify markers that were independently associated with urinary microalbumin levels. Serum creatinine and glucose levels were independent predictors of microalbuminuria. These findings are in agreement with another study [25] who found a positive association between hyperuricemia and diabetic nephropathy in comparison to those without diabetes, and this was attributed to the elevated glomerular filtration rate (GFR). Other literature also noted a positive association (*r* = 0.428, *P* = 0.001) of hyperuricemia with proteinuria and diabetic nephropathy [26]. The implication of measuring uric acid is that elevated levels may have a pathogenic role in the development of nephropathy rather than simply reflecting decreased renal uric acid excretion [25, 26].

Serum creatinine concentration is widely interpreted as a measure of GFR and is used as a marker of renal function in clinical practice [27]. Shemesh et al. [28] reported that in a reasonable proportion of patients with highly compromised GFR, serum creatinine concentration remained within the reference interval. Changes in muscle mass cause a variation in the creatinine pool independently of any GFR changes; thus, its dependence on muscle mass makes it an imperfect biomarker of GFR [27]. Obviously, this finding questions the value of serum creatinine, particularly in the early diagnosis of renal disease, and calls for caution when interpreting creatinine results. We recommend that if serum creatinine is to be used as a renal function marker among diabetic patients, it should be combined with microalbuminuria. This is primarily important in children and elderly diabetic patients who are likely to have lean muscle mass.

We observed a correlation between high glucose levels and microalbuminuria. Another study done by Bakris [4] noted that chronic hyperglycemia is a significant risk factor of diabetic nephropathy. Although microalbuminuria may be asymptomatic in diabetic patients, monitoring glucose levels and measurement of microalbuminuria should be carried out with the purpose of preventing end-stage renal disease (ESRD) among these patients. Roett et al. [29] supports the importance of glycaemic control as it plays a role in the prevention of progression to nephropathy.

Our study did not estimate urinary albumin/creatinine ratio (ACR) which is regarded as a reflection of albumin excretion rate (AER) [30] that can be measured in untimed spot urine samples. This is because urinary creatinine reflects muscular mass and so the latter may affect ACR. This means that low muscular mass can be a confounding in the use of urinary ACR to estimate AER. Indeed, [31] reported overestimation of microalbuminuria by urinary ACR in individuals with low muscular mass. We estimated AER using an immunoturbidity assay and found a significant relationship between serum creatinine levels (*P* = 0.006) and urinary microalbumin levels. Our findings corroborate the findings of Lutale et al. [32] who reported a significant correlation between serum creatinine levels and albumin excretion rate (AER) (*P* = 0.016). These findings provide basis for measuring microalbuminuria among diabetic patients in the study area.

We found no association between electrolytes and microalbuminuria. This is in contrast to what was reported by Kumari et al. [33] who investigated the association of serum electrolytes with renal function in DM. The authors found decreased serum sodium levels (hyponatremia) and increased serum potassium levels (hyperkalemia) to be statistically significant among diabetic patients who had high serum creatinine levels compared to those who had normal serum creatinine levels. This is likely to happen when there is hyperglycemia resulting in glucosuria, which is followed with hyponatremia. The authors concluded that electrolyte derangement is more with deteriorating renal function in DM.

We found sex and family history of DM to have a correlation with microalbuminuria at univariate analysis but only family history of DM remained statistically significant at multivariate analysis. The rest of the factors, which is, age, hypertension, systolic and diastolic blood pressure, duration of DM, alcohol consumption, smoking, and body mass index showed no correlation with microalbuminuria. In contrast to our findings, [20, 32] observed duration of DM, systolic blood pressure, and age as statistically significant risk factors of microalbuminuria. However, these studies considered Type 1 diabetic patients. Another study [34] found a significant correlation of microalbuminuria with duration of diabetes, and diastolic blood pressure. These studies seem to relate the duration of DM with microalbuminuria, a finding which is in contrast to what we report here. The difference may be because our study population had mostly adult patients above 45 years of age with overall average duration of DM being 6.8 years, pointing to Type 2 DM. The duration of DM in this study was determined from the year of diabetes diagnosis. Unfortunately, the exact time of onset of diabetes cannot be timed in most Type 2 DM patients since most patients seek health care later when symptoms of the disease become apparent. Thus, duration of DM in Type 2 diabetic patients should be interpreted with caution. It is interesting that in a study conducted by Alleyn et al. [21] on the impact of duration of Type 1 DM on persistent microalbuminuria, there was no difference among duration groups in the young children (8-12 years) while in older children and adolescent (13-18) years, persistent microalbuminuria varied significantly by the duration group. This variation in findings in the above studies may be due to clinical differences [21], genetics, definition of diabetes exposure, control of glucose levels, or lifestyle.

Logistic regression showed a weak association between microalbuminuria and estimated glomerular filtration rate (eGFR), but it was not statistically significant (eGFR), odds ratio = 1.2, 95% CI (0.24, 5.96), and *P* = 0.824. In a study done by Kalima et al. [35], decreased eGFR and microalbuminuria were noted as characteristic features of nephropathy. However, the weak association we observed in our study population should not be ignored because it can progress through the three developmental stages of diabetic nephropathy [20], and the patient finally ends up with ESRD, macroalbuminuria, and diminished eGFR [20]. End-stage renal disease is characterized with irreversible renal damage. Given that renal impairment especially in Type 2 diabetes is not always preceded by albuminuria and can occur in patients with normal eGFR [35], microalbuminuria together with traditional markers for nephropathy is needed to monitor renal function.

There were some limitations in this study. Firstly, it was limited to patients attending Mbarara Regional Referral Hospital. This being a referral hospital, it may have introduced a referral-bias and it would therefore be difficult to extend our findings to the general population of diabetic patients. Secondly, we used one random spot urine sample to determine microalbuminuria. Although this has been done elsewhere as noted above, some studies have recommended use of at least three measurements and confirmation is made if one shows microalbuminuria. Thirdly, it was not possible to precisely determine the duration of diabetes exposure, which we defined as the year when diabetes was diagnosed. Lastly, the cross-sectional nature of the study design limits the reliability of the observed associations between risk factors and microalbuminuria. A longitudinal study especially in the assessment of serum creatinine would be ideal. Despite these limitations, the substantial proportion of diabetic patients with microalbuminuria raises implications for future health policies.

## 5. Conclusion

The prevalence of microalbuminuria among diabetic patients at Mbarara Regional Referral Hospital was 22.9%. This prevalence is substantial considering that it can develop into macroalbuminuria and its associated complications. Monitoring blood glucose, creatinine, and uric acid levels even in the absence of reduced eGFR should be considered for patients with microalbuminuria.

## Figures and Tables

**Table 1:** Sociodemographic characteristics of participants.

Characteristic	*N* = 140 (%)
Sex	
Male	45 (32.1)
Female	95 (67.9)
Age categories in years	
18-34	13 (9.3)
35-44	25 (17.9)
45-54	41 (29.3)
55-64	36 (25.7)
≥65	25 (17.9)
Education	
Never went to school	18 (12.9)
Primary	76 (54.3)
Secondary	29 (20.7)
Tertiary (college/university)	17 (12.1)
Marital status	
Single	36 (25.7)
Married	104 (74.3)
Smoking	
Yes	3 (2.1)
No	137 (97.9)
Alcohol intake	
Yes	9 (6.4)
No	131 (93.6)

**Table 2:** Laboratory renal markers of the study population.

Characteristics *N* = 140	Mean (SD)	Reference intervals
Creatinine (*μ*mol/L)	109.8 (19.8)	74-127
Uric acid (*μ*mol/L)	245.8 (74.6)	214-488
Glucose (mmol/L)	9.3 (5.4)	3.9-6.4
Sodium (mEq/L)	153.8 (8.5)	135-155
Potassium (mEq/L)	4.7 (0.8)	2-7
Chloride (mmol/L)	98.4 (12.5)	97-108
eGFR (mL/min/1.73m^2^)	66.0 (14.8)	60-90
Normoalbuminuria (mg/dL)	0.68 (0.28)	<2
Microalbuminuria (mg/dL)	6.52 (5.05)	2-20
Macroalbuminuria (mg/dL)	33.14 (0.00)	>20

**Table 3:** Prevalence of microalbuminuria and its distribution according to age group and gender.

Prevalence type	*N*	% (95% CI)	*P* value
Microalbuminuria	32	22.9 (16.6, 30.6)	
Normoalbuminuria	107	76.4 (68.6, 82.8)	
Macroalbuminuria	1	0.7 (0.1, 5.0)	
Gender-specific			0.242
Male	13	28.9 (17.21, 44.26)	
Female	19	20 (13.03, 29.44)	
Age-specific			0.941
18-34	3	23.1 (6.32, 57.2)	
35-44	5	20 (8.0, 41.7)	
45-54	10	24.4 (13.3, 40.4)	
55-64	7	19.4 (9.2, 36.5)	
≥65	7	28 (13.2, 49.8)	

**Table 4:** Univariate and multivariate analysis for potential association showing the relationship between microalbuminuria and the traditional serum biomarkers.

Variable	Univariate analysis	*P* value	Multivariate analysis	*P* value
	Unadjusted coeff. (95% CI)		Adjusted coeff. (95% CI)	
Creatinine	0.010 (0.005, 0.014)	0.0001	0.006 (0.001, 0.011)	0.012
Uric acid	0.002 (0.001, 0.003)	0.0071	0.001 (0.0002, 0.002)	0.117
Glucose	0.030 (0.011, 0.048)	0.0017	0.027 (0.008, 0.0452)	0.005
Sodium	0.0002 (-0.011, 0.011)	0.9685		
Potassium	0.096 (-0.021, 0.213)	0.1067		
Chloride	0.004 (-0.003, 0.012)	0.2417		

## Data Availability

Data is available on request from the corresponding author of this article.
